# Corona and resource resilience—is efficiency still a desirable goal?

**DOI:** 10.1007/s00550-020-00493-2

**Published:** 2020-05-18

**Authors:** Mario Schmidt

**Affiliations:** Institute for Industrial Ecology (INEC), Pforzheim University, Tiefenbronner Str. 65, 75175 Pforzheim, Germany

The world will not be the same after the Corona crisis. But what does this mean for sustainability? Do established concepts need to be reconsidered? This article deals with the globally accepted concept of resource efficiency.

For many years, I have been concerned with the question of how companies can produce with a higher ecological efficiency and use fewer resources. I and my colleagues teach future engineers and managers that the efficient use of resources is an important component of sustainability. We have advised many companies and associations and have shown them how and where energy, material and environmental impact can be reduced^1^. The whole thing ran and runs under the heading of “resource efficiency”. The term has found its way into the scientific literature, is implemented in international standards^2^, in corporate practice and is now even included in national and international political programs, e.g. at the G7 or G20 summits^3^. But doubts are beginning to arise.

The orientation of our economic system and of our companies towards efficiency seems highly questionable in the times of the COVID-19 pandemic. In German we use the term: “auf Kante genäht”, which means “tightly sewn” and comes from the tailoring industry. It means that there are no safety margins that could be used if the garment has to be altered. That is what happens in the real economy at the moment: there are no sufficient reserves, no stocks, everything is oriented towards the principle of “just-in-time” in a globally networked economic system. If an important player fails, this may have a domino effect on entire supply chains. Redundancies in production and supply are missing. Is this a consequence of trimming all systems to efficiency, including eco-efficiency? Did the strategy of efficiency contribute to this supply situation?

Efficiency is commonly defined as the ratio between a certain benefit or result and the effort associated with it. The benefit is a social or economic quantity, which can be a product (“functional unit” is the term used in life cycle assessment), a service or simply the turnover in monetary terms. Effort can be measured in monetary terms, but also in terms of energy and raw materials used, emissions and polluted environment. The aim is to maximize the benefit, and of course to minimize the effort. This is the common classification of efficiency and it applies to both economic and ecological targets. Efficiency is often criticized in environmental circles because it does not impose an absolute restriction on emissions or pollution, but is always in relation to the benefit. If the benefit is increased, the cost increases as well, even if the measure is efficient. But that is a different discussion, which should not be the subject of this article.

Winfried Kretschmann, the green Minister President of the German state of Baden-Württemberg, expressed it this way: “Although resource efficiency does not automatically imply sustainability, sustainability is inconceivable without resource efficiency. That is because it is all about using only as much as is necessary to achieve a desired result.”^4^ I think this sentence is still true. If I am not efficient, then I am wasting something. Products or materials may end up in waste or in nature without having served any purpose. This has been a recurring theme in recent years, for example, globally around 14% of the world’s food is lost from production before reaching the retail level^5^. In many world regions the water supply is also highly inefficient, with much of the drinking water being lost along the way in inadequate supply structures. These inefficiencies must be eliminated. They have no benefit. They are avoidable from a technical or organizational point of view, and only occur because those who are responsible have not fulfilled their tasks optimally. In this respect, efficiency is still required.

What is more decisive is the question of purpose, which must be reconsidered. The concept of effectiveness could also be linked to this: What is the use of efficiency if the purpose is wrongly chosen? This is precisely what effectiveness expresses, namely doing the right thing.

Until now, the purpose has been to gain as much return as possible per input of raw materials, labor, capital or money. But it now becomes clear that a purpose must also be to create reserves for bad times or for catastrophes—actually an old biblical wisdom. It has been lost in these fast-moving times and in view of the global and rapid availability of everything, of goods, money and information. The Germans, for example, are world champions in insuring themselves against all kinds of risks, which makes them feel safe and costs their money. But they could also have spent the money on real physical safety stocks. After all, the insurance money is useless if respirators, disinfectants or, ultimately, even food is missing.

Efficiency is still required, but we need to think more encompassingly about the purpose of our economic activities. After all, the benefit is more than just the short-term EBIT of a company; it is the long-term livelihood and reputation of the company, and the benefit it generates for society. This can also include always being able to deliver, to supply sufficient food, spare parts or protective equipment when needed, even if the stockpiling required for this is not profitable for many years. But this determines how flexibly a system can react to new challenges or crises. Of course, this so-called resilience has its price.

The question of resilience also arises in the area of resources. Meanwhile, China is the main player in supplying the world with important raw materials. The German Federal Institute for Geosciences and Natural Resources points out that 18% of the world’s mining activity today takes place in China. More than 50% of the refined products (measured by monetary value) come from China^6^. This should not be misunderstood: It is right and proper that China is engaged in this area and is playing an important role in the global economy, even if it would be desirable that more ecological and social standards were to be met. But what if such a state temporarily fails due to a crisis like the virus pandemic? Even for China, it cannot be desirable to have no backup in other countries to rely on in crisis situations. Greater attention must be paid to the global diversification of production and also of the supply of raw materials. Redundancies are necessary, and therefore they are not inefficient, but must be seen as an additional benefit—not as unnecessary effort.

Germany and other industrial nations take great care to ensure that no monopolization of individual companies takes place, as this could negatively affect the market and the consumer, e.g. lead to higher prices. But who ensures that there is not too much dependence on other countries, regions or technologies? In Germany, for example, the recycling of metals is seen as a strategy to reduce the primary mining of raw materials and the associated environmental pollution. In some cases, recycling is even seen as a contribution to reduce Germany’s dependence on raw materials from other countries. But not enough is done to ensure that industrial processing capacities are also made available in Germany or Europe, for the number of refineries and metal smelters is decreasing, partly even because environmental regulations are becoming more and more stringent. Instead, Germany relies on capacities in other countries, some of which have much poorer environmental standards and belong to political or economic risk areas. This helps neither the environment nor the supply reliability, but is simply wrong ecological activism.

Therefore, resource efficiency will continue to be an important issue and an important component of sustainability strategies in the future. However, one should add resource resilience to the term, and this applies not only to energy or metal ores, but also to natural resources such as water or biodiversity. Here, safety margins are necessary; the use of these resources must not be too “tightly sewn”.

As far as the core of the efficiency strategy is concerned, the question is not what is the effort, but what is the benefit. For example, do we need national or transnational raw material storage facilities for crisis situations? Up to now, such facilities have only been established for military and energy reasons.

Both industrial policy and environmental policy must ask how technical and geographical monopolies can be avoided. Not only for reasons of economic policy, but also—as we can see at present—for health and ecological reasons. Which industrial and metallurgical infrastructure is necessary in Germany or Europe to ensure the long-term supply of raw materials on the one hand, and to be able to ensure high-quality production on the other, and not to be dependent on dubious ecological and social production conditions in distant countries?

And, of course, the question arises which production and trade structures, which industrial redundancies are necessary in order to maintain the supply even in crisis situations. None of this speaks against efficiency, but rather for a reassessment of what we consider as a benefit to society. Thus, we should also see the resilience of our systems as a valuable benefit.

## Footnotes

1 e.g. Schmidt, M. et al. (2019): 100 Pioneers in Efficient Resource Management. Best pratice cases from producing companies. Springer Berlin/Heidelberg.

2 e.g. the ISO standard 14045 about eco efficiency assessment, the ISO standard 14051 about Material Flow Cost Accounting, the standard of the German Association of Engineers VDI 4800 about Resource Efficiency.

3 Leaders’ Declaration G7 Summit in Germany Schloss Elmau, 7–8 June 2015, p. 17; G20 Leaders’ Declaration in Germany Hamburg, 7–8 July 2017, p. 12.

4 Cited in Schmidt, M. et al. (2019), p. V.

5 Food and Agriculture Organization of the United Nations (FAO) (2019): The State of Food and Agriculture 2019. Moving forward on food loss and waste reduction. Rome.

6 Steinbach, V. (2020): Assessment of risks in raw material supply chains—from conception to implementation (in German), Berliner Recycling- und Sekundärrohstoffkonferenz, 2. und 3. März 2020, Berlin.

